# What distinguishes data from models?

**DOI:** 10.1007/s13194-018-0246-0

**Published:** 2019-01-15

**Authors:** Sabina Leonelli

**Affiliations:** Exeter Centre for the Study of the Life Sciences (Egenis) & Department of Sociology, Philosophy and Anthropology, University of Exeter, Byrne House, St Germans Road, Exeter, EX4 4PJ UK

**Keywords:** Data model, Experimentation, Empiricism, Research practice, Plant science, Data processing, Statistics, Phenomics, Big data, Inference

## Abstract

I propose a framework that explicates and distinguishes the epistemic roles of data and models within empirical inquiry through consideration of their use in scientific practice. After arguing that Suppes’ characterization of data models falls short in this respect, I discuss a case of data processing within exploratory research in plant phenotyping and use it to highlight the difference between practices aimed to make data usable as evidence and practices aimed to use data to represent a specific phenomenon. I then argue that whether a set of objects functions as data or models does not depend on intrinsic differences in their physical properties, level of abstraction or the degree of human intervention involved in generating them, but rather on their distinctive roles towards identifying and characterizing the targets of investigation. The paper thus proposes a characterization of data models that builds on Suppes’ attention to data practices, without however needing to posit a fixed hierarchy of data and models or a highly exclusionary definition of data models as statistical constructs.

## Introduction

This paper investigates the relation between data and models, their respective roles as research components within empirical inquiry, and the reasons why these roles should be kept distinct within scientific epistemology. I focus on the epistemic function of data models and the circumstances under which they should be distinguished from data. The account is developed through a detailed reconstruction of the stages of data processing involved in contemporary plant phenotyping and specifically the use of high-throughput imaging data to acquire insight into plant development and growth patterns – a case which is representative of exploratory research practices within the life sciences (and beyond), and yet has received next to no attention from philosophers of science.^1^ This enables me to highlight philosophically significant aspects of the activities of data production, processing and interpretation, and argue that whether a set of objects functions as data or models does not depend on intrinsic differences in their physical properties, level of abstraction or the degree of human intervention involved in generating them, but rather on their distinctive roles towards identifying and characterizing the targets of investigation. I thus use the analysis of data practices as an entry point into the study of data modelling and inferential reasoning whose applicability extends well beyond the case under consideration.

This is not a completely new approach to the study of modelling, as exemplified by Patrick Suppes’ seminal account of the hierarchy of models, which was itself grounded on an analysis of the processes through which researchers go from data collection to the formulation of theories and the crucial role played by models in enabling that shift (Suppes 1962). In what follows, I take inspiration from Suppes’ approach and scrutinize the ways in which data and data models are generated and used within contemporary science. In contrast with Suppes however, I consider research practices where some of the data being modelled come in forms other than numerical (namely, as images); and where statistical analysis is coupled with: qualitative judgements around what data to consider for further analysis, shifts in what objects are actually considered to be data, and the implementation of computational tools to extract measurable traits from images in an automated fashion.^2^ In such a case, which is often encountered especially within the biological, social, environmental, historical and health sciences, Suppes’ hierarchy of models proves difficult to apply and does not help to resolve questions around the nature and epistemic function of data and data models.

To make better sense of the variety of data practices found across the sciences, I propose to move away from a structural characterization of data models altogether and instead to distinguish data from models by virtue of the circumstances and purpose of their use within situations of inquiry. I argue that many of the operations through which researchers process data are primarily aimed towards making them useable as evidence for claims, whether or not the specific targets of the claims in question have been clearly defined – and thus, in Bogen and Woodward’s terms (1988), whether or not data are endowed with the power of representing one or more phenomena. In deciding what counts as useable data, researchers define the evidential scope of their investigation, that is the range of phenomena that they will be able to consider once they start clustering and ordering data in ways that may help to interpret them as evidence. The clusters of data thus obtained (which may take various forms, depending on which visualisations researchers find most tractable as evidence) are what I call data models: that is, arrangements of data that are evaluated, manipulated and modified with the explicit goal of representing a phenomenon, which is often (though not always) meant to capture specific aspects of the world.^3^ Hence data models are defined by the representational power that researchers impute to them, and play an essential role in specifying the target of the claims for which data can be used as evidence – in other words, the phenomenon being investigated.

The argument is structured as follows. In the second section, I introduce what I call the representational view of data and models, and highlight the difficulties generated by this widely held view when attempting to analyse the distinctive epistemic roles of these research components. In section three, I discuss existing scholarship on data models, where questions about the relation between data and models have been most closely addressed. I trace the motivations underpinning Suppes’ seminal work and note his commitment to highlighting and defending the significance of statistical reasoning within knowledge production. While this commitment has proved generative to philosophers and researchers concerned with formal methods of inquiry, I note that it takes attention away from aspects of data processing and inference that are not informed by statistical techniques. In section four, I delve into my case study and reconstruct data practices involved in the experiments carried out at the National Centre for Plant Phenotyping (NCPP) in Aberystwyth in Wales. In particular, I focus on the SureRoot project - a collaboration between the NCPP and the North Wykes Farm Platform in Devon, England that was carried out between 2014 and 2017 to improve understanding of root systems.^4^ My analysis focuses largely on the part of SureRoot that was developed at the NCPP, within which I identify seven distinct stages of data processing, each of which involves distinctive research skills, interests, assumptions and decisions.^5^ The case exemplifies the diversity of expertise involved in data processing activities and the specific challenges linked to exploratory research. Section five examines the role that representational assumptions play within each stage and problematises the idea of representation as sole or even primary epistemic goal for the researchers involved. In closing, I consider the implications of this analysis for understanding the relationship between data and models, the crucial role that data models can play in identifying the targets of scientific investigation, and the epistemology of empirical inquiry more generally.

## Data and models as representations

The nature and epistemic role of data remain under-researched topics in philosophy of science, especially when compared to the extensive scholarship on models and modelling activities. Philosophers tend to assume that data have some sort of representational content, in the sense of instantiating some of the properties of a given target of investigation in ways that are mind-independent. This representational conceptualization of data epistemology is often viewed as playing an important role in understanding the empirical basis of scientific knowledge, since the properties instantiated by the data are the medium through which the world, in its unpredictable complexity, becomes amenable to scientific study. Data are taken to capture and convey the same information about the world regardless of the circumstances of inquiry, and particularly of the assumptions and background knowledge of the researchers who are using them as evidence; such assumptions may colour the extent to which researchers are able to extract information from data, but do not affect the content of data as documents of specific aspects of reality. Hence, the informational content of data is regarded as fixed and context-independent. In such a framework, statistical analysis plays a crucial role in guaranteeing the reliability of data and the validity of the inferences extracted from them.^6^ What data suggest about the world can of course be misunderstood and misinterpreted whenever researchers use the wrong inferential techniques or modelling approach, but data themselves are viewed as reliable information sources – a mere “input” into processes of modelling. Finding the right interpretation involves finding the right tools to extract truth from data.^7^

**Fig. 1:**
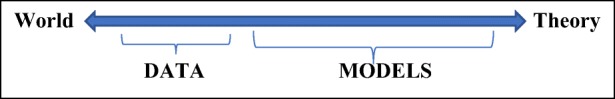
A graphic rendition of representational view of data and models, with the spectrum between world and theory standing for what is being represented, and data and models indicating what representations are associated to which parts of the spectrum

A key problem of the representational view of data is that it makes it hard to differentiate data from models, given that models are themselves typically conceptualised as representations – though what they represent can vary from (parts of) the material world to highly abstract concepts. Miles MacLeod and Nancy Nersessian (2013), for instance, describe model-based reasoning as “a process of generating novel representations through the abstraction and integration of constraints from many different contexts (literature, target, analogical source, modeling platforms and so forth)” – a view they broadly share with Daniela Bailer-Jones (2009), Mauricio Suarez (2004), Michael Weisberg (2013) and Alex Gelfert (2016), among many others. Even authors who emphasise the use of models as tools – “mediators” or “artefacts” – whose chief research function is to enable interventions, such as Knuuttila (2011) and Mary Morgan (2012), note the value of models in “standing for” specific phenomena. Data are not given much prominence within these accounts, with most authors treating them as empirical input for modelling. This can be interpreted as implicitly accepting a view of data as intrinsically reliable representations of the world. And indeed, while many philosophers have no trouble recognizing models as representations that may well be fictitious or false and yet yield “true theories” (e.g. Wimsatt 2007; Toon 2012), there is strong resistance against treating data in the same way and some confusion around what it is about data that gives them the epistemic power to provide empirical warrant and even direct support for claims about phenomena.^8^ It could be argued that within these accounts, data and models exist on a representational continuum between theory and the world, with data typically taken to operate closer to the “world” end of the spectrum and models to the “theory” end [see Fig. 1].

This is an uncomfortable position for philosophers to be in. Ronald Giere (1999) signals that discomfort when conceptualizing scientific research as “models almost all the way down.” One the one hand, that position stresses the constructed and theory-laden nature of the objects used by scientists to investigate and represent the world, whether they be concepts, diagrams, maps, equations or material objects such as a scale model. On the other hand, Giere is at pains to point out that data is somewhat different from the various representations created by scientists to make sense of reality: data may be theory-laden, but are still the closest one gets to an objective document of scientists’ interactions with the parts of the world that they study, and thus need to retain properties that would make them adequate and credible empirical grounding for claims about phenomena, irrespectively of the representational value that researchers choose to bestow upon them. Similarly, in their seminal paper on data and phenomena Bogen and Woodward (1988) rightly emphasize the situated and deeply embedded nature of experimental data, thus following in the long philosophical tradition arguing against the existence of “raw data” providing unmediated access to reality. However, this leaves them struggling when pointing to the importance of data as ultimate arbiters of empiricism.^9^ Just like Giere, they provide an argument for *why* we should avoid a view of science as “models all the way down”, but do not offer a view on *how* this can be achieved.

## What are data models? Problems with Suppes’ account

**Fig. 2:**
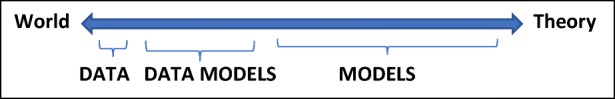
The place of data models in the representational view of data and models depicted in Fig. 1

The closest that philosophers have come to explicitly discussing the relation between data and models is through consideration of what Suppes called “data models”. Within the representational account described in the previous section, these models consist of an intermediate step between data and models (Fig. 2).

This makes data models into an excellent starting point towards investigating how data relate to models, and yet does not by itself resolve fundamental questions around the status of data in the representational spectrum, nor does it help to offer an account of how data may operate differently from models and hence be reliably used as sources of empirical evidence for the models themselves. The contemporary characterisation of data models as “corrected, rectified, regimented and in many instances idealized version of the data we gain from immediate observation, the so-called raw data” (Frigg and Hartmann 2016) demonstrates how the very distinction between data models and other types of models is predicated upon presupposing the existence of “raw data” resulting from “immediate observation” of the world, and thus arguably providing direct and unmediated access to it.

A key motivation for Suppes’ examination of how modelling practices relate to data production activities was precisely the recognition of this vicious circle and its troubling implications for philosophical accounts of what it means for scientific research to be empirically grounded. Suppes was deeply concerned with the complexity of data processing activities within experiments, and it was the study of the means and motivations used for procedures such as data reduction and curve fitting that inspired him to differentiate between models of theory, models of experiment & models of data. In his words, “the exact analysis of the relation between empirical theories and relevant data calls for a hierarchy of models” (Suppes 1962, 33). This was not, however, the only motivation behind Suppes’ account. As he made clear when first presenting the notion of data models, an equally powerful goal was to “to show that in moving from the level of theory to the level of experiment *we do not need to abandon* formal methods of analysis” (ibid., 260; see also Suppes 2007). Indeed, Suppes was so concerned by what he called the “bewildering complexity” of experimental situations, that he worried that philosophers would not appreciate the ways in which statistics can and does help scientists to abstract data away from such complexity. This concern motivated his choice to further distinguish between models of data and a large group of related research components and activities used to *prepare* data for modelling, which include models of experiment (which describe choices of parameters and setup) and practical issues such as sampling, measurement conditions, and data cleaning. Suppes describes these “pragmatic aspects” as encompassing “every intuitive consideration of experimental design that involved no formal statistics” (1962, 258), and depicts them as the lowest steps of his hierarchy – at the opposite end of its pinnacle, which are models of theory.^10^

My worries with Suppes’ characterisation stem not from these distinctions per se, but rather from his conclusion that “once the empirical data are put in canonical form, every question of systematic evaluation that arises is a formal one” (ibid, 261). In other words, Suppes concluded that once data are adequately prepared for statistical modelling, all the concerns and choices that motivated data processing become irrelevant to their analysis and interpretation. Thus, Suppes argued that data models are necessarily statistical models, that is objects “designed to incorporate all the information about the experiment which can be used in statistical tests of the adequacy of the theory” (Suppes 1962, 258). His formal definition of data models reflects this decision, with statistical requirements identified as the ultimate criteria to identify a data model and evaluate its adequacy: “Z is an N-fold model of the data for experiment Y if and only if there is a set Y and a probability measure P on subsets of Y such that Y = <Y, P> is a model of the theory of the experiment, Z is an N-tuple of elements of Y, and *Z satisfies the statistical tests of homogeneity, stationarity and order*” (1962, my emphasis).

Many philosophers have accepted and further promoted Suppes’ decision to define data models as statistical models. Prominent examples range from Deborah Mayo, who in her seminal book *Error and the Growth of Experimental Knowledge* asked: “What should be included in data models? The overriding constraint is the need for data models that permit the statistical assessment of fit (between prediction and actual data)” (Mayo 1996, 136)^11^; to Baas van Fraassen, who despite holding different views on the nature of science from Mayo, also embraced the idea of data models as “summarizing relative frequencies found in data” (Van Fraassen 2008, 167). Through works such as these, Suppes’ legacy has come to be identified with the focus on statistics as an essential component of data modelling, thus underestimating his broader concerns with the epistemology of data and his curiosity about experimental practices where such formal approaches to inferential procedures from data are not readily applicable or even relevant.^12^

I want to argue that the insistence on formal methods as an entry point into the analysis of data processing which characterizes Suppes’ work and much of contemporary philosophy of science fails to tackle critical questions around the source of the epistemic value of data, and the relation between data and models. This is, first, because this analysis deals only with a subset of the objects that scientists working across different fields identify as “data”: that is, those objects – typically numbers or symbols - that can be subjected to statistical manipulation. This precludes Suppes’ approach from being applied to research situations where data are not quantities that are amenable to statistical treatment, and/or where statistical methods of analysis are not used as a means of validating data models, but rather as a way to visualize data (for instance by helping to arrange data into graphs, as illustrated below) – not to speak of cases where statistical methods are not used for data analysis at all. Second, it is hard to see how Suppes’ views can apply to cases where what research questions are being investigated, which conditions are ceteris paribus, and what constitutes the target phenomenon, are not given at the start of the inquiry – as is typically the case within exploratory research. Third and perhaps most important, Suppes’ approach makes uncritical assumptions about the ease with which researchers can identify “raw data” and dismisses the tight intertwinement between activities of data acquisition and data manipulation. As Todd Harris has shown in relation to data models in physics, “in many cases the data that has traditionally been referred to as raw is in fact a data model”, an observation from which Harris concludes that “the process of data acquisition cannot be separated from the process of data manipulation” (Harris 2003).

In what follows, I build on Harris’ analysis and expand on its significance by considering a case of data processing where the differentiation between data models and “simple datasets” is indeed problematic, particularly when it is approached as a difference in the physical characteristics of these research components. I show that researchers can and do change what they consider to be “raw data” to suit different investigative purposes, resulting in changes to the informational content attributed to data and thus their value as evidence for claims. More broadly, I aim to provide an alternative to Suppes’ account of data models that (1) does not rely on problematic and fixed assumptions about what “raw data” need to be; (2) can be applied to cases of exploratory research and situations where statistics is not central to data analysis; and (3) addresses and resolves the problem of distinguishing data from models, by defining both research components through their relation to inquirers and their role within specific epistemic activities.

## Stages of data processing: A case from plant phenotyping

Phenotyping is the area of the life sciences devoted to the study of morphology at all levels of biological organization, ranging from the molecular to the whole organism, under varying environmental conditions. A long-term component of botany, phenotyping is currently undergoing a revival within plant science, where it is recognized as crucial to the analysis of gene-environment interactions.^13^ For instance, phenotyping is indispensable to understanding how shoots and roots respond to drought or flooding – which in turn informs estimates of the impact of weather conditions associated to climate change on agricultural yields, thus facilitating the development of what researchers call “precision agriculture” to tackle the urgent social challenges associated with food security. A recent review in Plant Methods describes phenotyping as a “quantitative description of the plant’s anatomical, ontogenetical, physiological and biochemical properties” (Walter et al. 2015). One of the key challenges in this field – and the reason for choosing it as a case study to illustrate my argument - is precisely the transformation of complex qualitative objects such as free-text descriptions and images into machine-readable data that can be subjected to computational analysis. Contemporary phenotyping relies heavily on the analysis of large sets of imaging data, which are produced at a fast rate and high volume through automated systems comprising several cameras, each geared to capture different signals (ranging from the visible to the infrared spectrum of light; e.g. Fahlgren et al. 2015). As this section illustrates, efforts to find ways of analyzing these data are deeply intertwined with efforts to develop tractable specimens, instruments and computational tools, a complex set of iterations and expertise that defines not only how plants are described, but the type of questions and phenomena that researchers end up focusing on.

The “roots for the future” (SureRoot) project provides a good instance of the challenges involved in processing phenotypic imaging data. The goal of the project was to understand grass-soil interactions in order to improve root strength, depth and ability to efficiently use water.^14^ This was achieved in two steps. Part A, which is what I will focus on here and was carried out at the NCPP, generated and analyzed a vast set of root imaging data in order to assess how root structures linked to specific genetic traits fit different soil conditions. This involved relying on the tightly controlled climatic and experimental conditions characterizing the “smart glasshouse” of the NCPP, within which plants are carefully monitored and regularly photographed through the use of conveyor belts set up to bring the plants to five different imaging chambers as often as required (sometimes multiple times per day, to capture fine-grained patterns of plant development).^15^ Part B, which was carried out at an experimental farm where plant specimens were grown in full exposure to the natural environment, aimed to generate comparable field data, with the purpose of checking the external validity of results obtained on plants grown under more controlled conditions.

During a research visit to the NCPP in 2015, I identified seven stages involved in the production and processing of data within part A of the SureRoot project, which I briefly discuss below.

### Stage 1: Preparing specimens

The first requirement towards the production of useable imaging data is to grow plant specimens that are amenable to the transport and imaging technologies employed in the smart glasshouse. While the initial parameters for the experiment are provided by plant scientists, including the choice of which species to use (in this case, the tall grass *Festulolium*), it falls mostly to the technicians that run the glasshouse, the adjourning fields and the imaging facilities to ensure that specimens satisfy the requirements of experimental design. A considerable amount of care and know-how is required to plant seeds so that they are equally numbered and spaced in every pot and maintain the growing plants so that their size and growth rate stays within a range that makes imaging results comparable across plants. The health of plants is also carefully monitored, with plants that manifest unusual traits marked out as unusable and/or potentially interesting for other investigative purposes (such as understanding whether the trait is the result of a mutation or environmental exposure – a point to which I shall come back below). This kind of standardisation, which I have elsewhere discussed as a form of material abstraction used to create material models (Leonelli 2008), is particularly complex to achieve in this case since the experiment requires growing plants on real soil, which is itself a source of variability given its highly diverse microstructure and mineral and microbial composition.^16^ Further elements to keep under control are the conditions under which plants travel to the imaging chambers. Conveyor belts are not fully reliable, with sudden jerks resulting in plants being thrown off (and thus a gap in imaging data) or dirty pots (that damage the extent to which images can be compared). Plants themselves also play tricks on the technology by shedding leaves which can jam the conveyor belt (problematic especially overnight, when humans are not at hand to check) and/or compromise the comparability of the images.^17^ Finally, environmental controls can also fail in ways that researchers had not predicted or accounted for. Six months after the inauguration of the facility, for example, NCPP technicians realised that in some of the experiments being carried out, the temperature difference between the glasshouse and the imaging chambers was giving plants a thermal shock, a factor which may affect the measurement of plant temperature responses. Such issues are amplified when imaging plants that are grown outside the glasshouse.

### Stage 2: Preparing and performing imaging

**Fig. 3:**
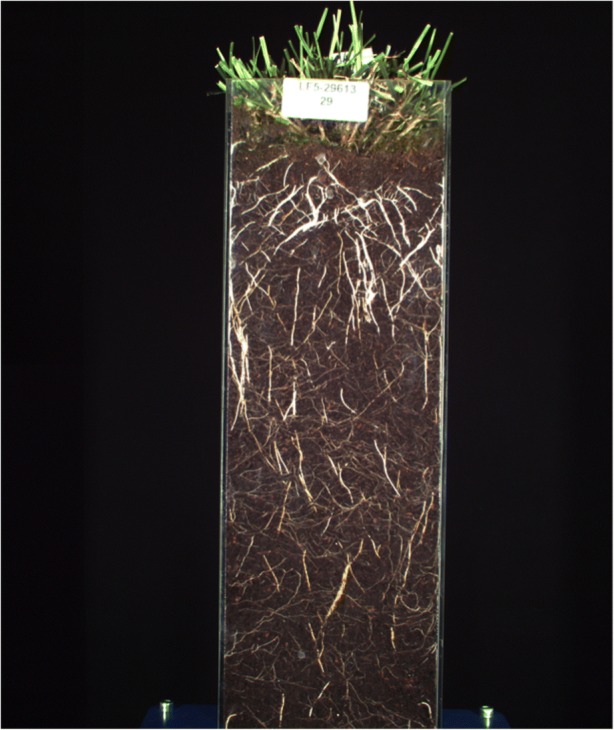
Example of Festulolium plant image produced for SureRoot

Another key condition for the experiment is identifying appropriate conditions, techniques and tools for generating digital images. Technicians consider the desired background, resolution, focus, lightning conditions and angle of the pictures, as well as the number and interval of repeats per plant – which is constrained by which imaging tools are employed and how they are calibrated, as well as the number of experiments to be carried out at any given time^18^ – and what counts as ‘dirt’ and ‘debris’.^19^ Technicians also develop techniques and tools such as glass pots to make the roots visible to imaging, and imaging specialists are consulted on how to adapt available imaging techniques and instruments to the experimental conditions of the glasshouse. The result are images such as Fig. 3 below. Generating such an image involves a vast amount of know-how that affects the extent to which the resulting data are viewed as “usable” by researchers, and yet is not typically recorded systematically. In the words of a technician, “quite often that sort of stuff is lost and it stays in somebody’s lab book, or in their computer, or on their server, but, in essence, that person moves on and the group goes to do other things. It could be recreated through an immense amount of work. It’s almost as bad as [..] stuff being lost in breeders’ notebooks. It’s the modern equivalent of that” (PI_1_C).

### Stage 3: Data storage and dissemination

The third stage of data processing involves storing and labelling the images being produced, so that they can be searched and retrieved as required for analysis. I have shown in previous work the epistemic importance of this stage and the challenges and tools involved in organizing data collections so that they can be easily searched and used for specific investigative purposes (Leonelli 2016). This is where data managers step in, bringing expertise on available systems for the curation and classification of data and related contextual information (“meta-data”). This work involves interpretative decisions around which types of inquiry the imaging data could help to address. In the case of SureRoot, it is clear that the images can serve as evidence for claims about roots, and they can therefore be classified under that term. However, depending on how they are subsequently analyzed, the images could provide a host of other information about the plants, for instance about stem and leaf growth. By labelling data with reference to which phenomena they may be used to document, data managers contribute to identifying and circumscribing their evidential value in ways that shape their usability for analysis. The same holds for decisions around how to label meta-data (documenting for instance plant provenance and growth conditions), which determine how researchers evaluate the potential significance of data and what they can be taken to represent.^20^ Last but not least, and typically in consultation with biologists and technicians, data managers control access to the data, by deciding whether and how the data are shared among NCPP staff, external collaborators and other stakeholders – a decision that can have significant implications for which data formats, labels, software and visualization tools are ultimately chosen to carry the data.^21^

### Stage 4: Coding for image analysis

**Fig. 4:**
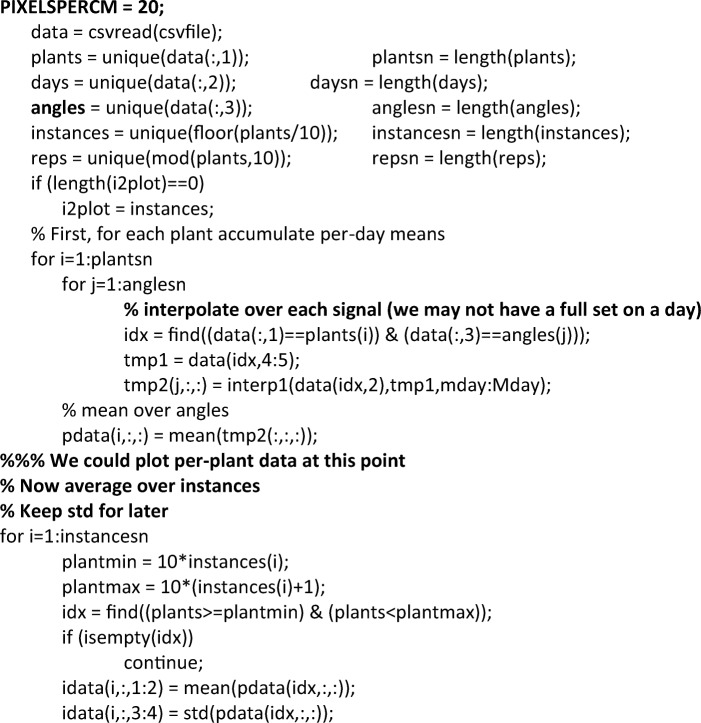
Section of the code used to analyse root width data, with notes inserted by computer scientists upon my request to highlight their adaptations. In this code, the programmers have (1) defined the ideal granularity of image analysis; (2) refocused the programme on angles at which roots typically grow; (3) decided on temporal segmentation (signals per day, corresponding to number of measurements); and (4) calibrated for potential absence of regular signals

The fourth stage involves developing software that can support the analysis of imaging data. This is where computer scientists enter the fray, initially consulting with biologists about the aims of the experiment, but then working largely on their own to develop a programme through which images could be mined. This process includes evaluating which measurements could be effectively extracted from the imaging data through computational means, so as to make it possible to accurately and consistently compare root systems. In this case, the task was overseen by a senior, highly skilled computer scientist with decades of experience, who discussed with me the difference between his approach and the biologists’ in the following terms: while the latter look for ways to use data to answer biological questions, often resulting in chasing methods to capture information that was hard to extract from the available files, the former focus on identifying information that could be easily harnessed with available computational tools - whether or not such information had immediate biological significance. Rather than focusing on the biological questions at hand, computer scientists thus approached the questions of how to analyse the plant images by considering which properties of the images at hand would be most easily and reliably amenable to analysis through existing computational tools. As a result of this research, computer scientists zoomed on measurements of root width, number and positions within the pot, which could be analysed by tweaking an existing programme available within the widely used software MatLab so that it would capture plant-relevant parameters. Tweaks included determining the range of expected minimal and maximal width of roots, their geometrical angles and location, the required spacing of pot image, the relevant time intervals and the number of measurements per day (Fig. 4).

### Stage 5: Image filtering

**Fig. 5:**
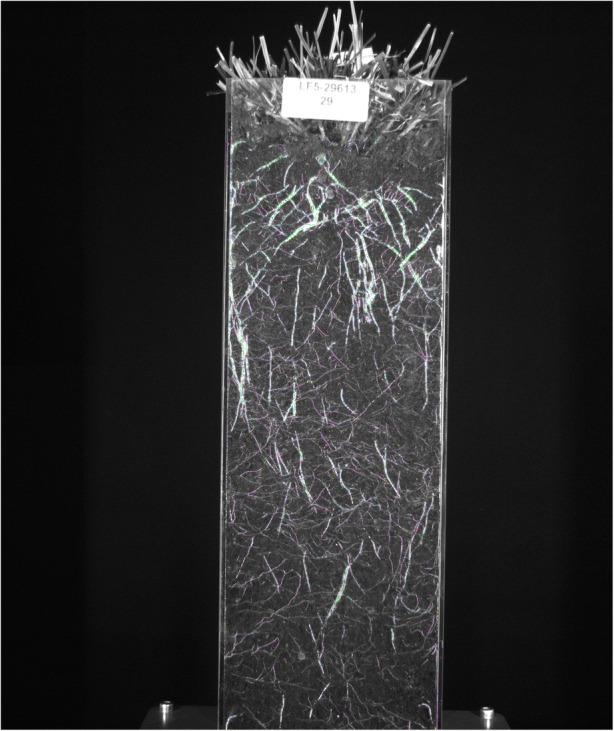
Image from Fig. 3 filtered through the code developed in stage 4

Once satisfied with the code, the computer scientists used it to analyze thousands of plant images, resulting in a series of “filtered images” that look much like the original data to the untrained eye (see Fig. 5), and yet have been modified in order to make the parameters of the analysis more prominent and easier for the computer to pick up, while features that are considered to be less significant (such as very small roots positioned at awkward angles) disappear from the image.

### Stage 6: Image analysis

**Fig. 6:**
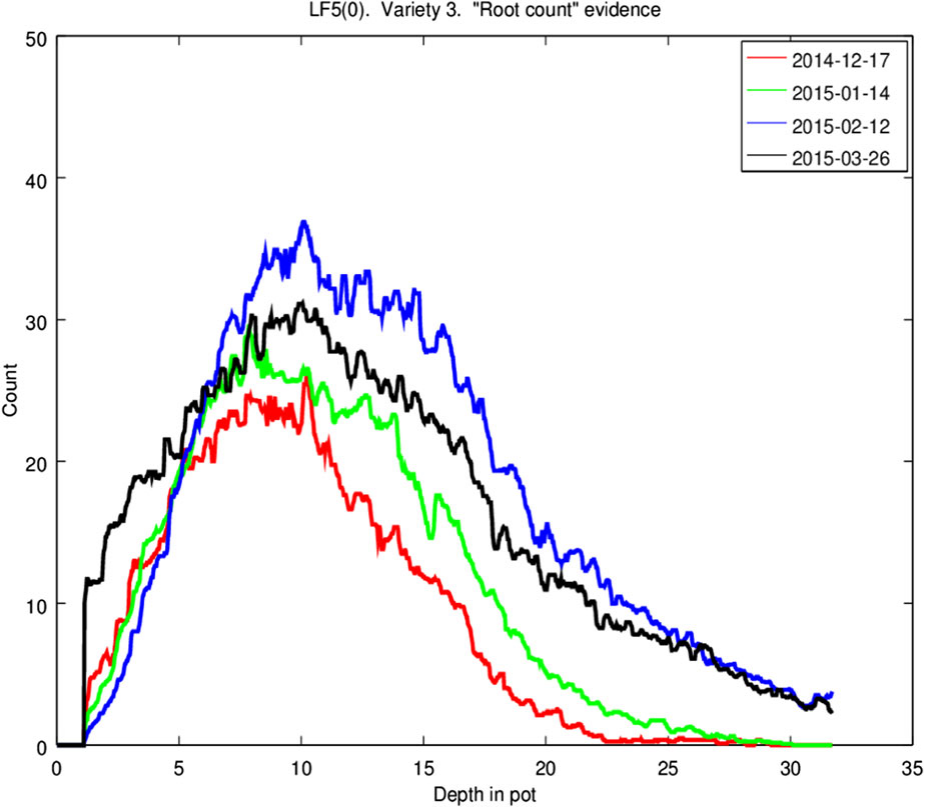
Example of a plot graph obtained through the computational analysis of filtered imaging data, juxtaposing the average number of roots and their depth and comparing the results of four experimental runs

Now it was possible for the computer scientists to automate the extraction of parameters from each filtered image, so as to produce plot charts that quantified and analyzed root distribution relative to soil structure and depth of roots in pot (e.g. Fig. 6). Again, there are computational constraints on what kinds of analysis and graphical representations can be selected to visualize the data at this stage, and the computer scientists playing around with the design and color of visualizations and the ways in which the coding and filtering affect the charts. Computer scientists emphasize the idea of “play” as crucial to their work, especially when compared with the work of biologists and technicians. For one thing, playing around with images is cheaper than running experiments, with more trial and error allowed; and “data games” can be interrupted, picked up again, interspersed with other tasks without significant disruption. More importantly, “sometimes the problems phenotyping poses are not amenable to AI/vision attacks. Sometimes the fun we can have writing programs is of no relevance to phenotyping. But we think the interesting solutions are often to problems that were not set. Interdisciplinarity can be a difficult exercise in rephrasing what is wanted in language the other people can understand” (R_3_A). This interdisciplinary game works precisely because it is not subject to constraints around what kind of knowledge would be valued or even accepted by biologists, and yet it has a decisive impact in shaping what phenomena biologists ended up targeting within the project, and what the imaging data were taken to be evidence for.

### Stage 7: Calibration and further analysis

The seventh stage of data processing is where biologists finally take the center stage. They consider the biological significance of the plot charts in light of what is known about the target system in question, and propose variations in parameters, sampling, image analysis and so forth in order to enhance the usefulness of the plot charts as evidence for specific knowledge claims. This process involves biologists re-defining and narrowing their research questions, so as to fit the data at hand; and revisiting previous stages of data processing to understand how the data came to be selected and visualized in this way, which can lead them to query decisions previously taken by technicians, imaging specialists or computer scientists. In some cases, such as for instance the width of roots to be taken into consideration for analysis, biologists’ questioning can lead to modifying the decisions taken at one or more stages of data processing, in the kind of iterative feedback loop discussed by Hasok Chang (2004). In other cases, the decisions taken by non-biologists turn out to constitute impassable constraints, either because of the effort and time it would take to do things differently or because of the physical properties of the data and related materials, equipment and infrastructure (ranging from imaging chambers and cameras to the servers, databases, software and plant specimens themselves). The data plots that biologists end up accepting as outputs of the imaging experiments and use as empirical basis for their interpretation (i.e. the outcome of stage seven) are thus the product of a large variety of skills, motivations and types of background knowledge, many of which have more to do with the conditions under which data are handled than with considerations about the biological meaning of the data – or, in other words, the extent to which the data represent specific features of plant biology.

In the next section, I discuss four philosophical lessons that are vividly exemplified by this case.

## Lessons learnt: Distinguishing modelling from data processing

### What counts as data can change at every step of inquiry

We have seen how each stage of data processing involves a different ensemble of expertise, skills and goals. Technicians and image specialists are primarily interested in setting up and maintaining plants, equipment and the experimental environment so as to produce *adequate specimens and images* without mistakes, break-downs or inconsistencies in controls. Computer scientists aim to deliver *efficient solutions for the automated analysis* of complex images. Data managers want to select and develop protocols, standards and infrastructures to *store and disseminate data* so that the data are easily retrievable and re-usable for as wide a variety of investigative purposes as possible. Biologists aim to *set up experiments* and *analyze* data in ways that define and inform research questions, help identify and characterize phenomena of relevance and ultimately teach them something about the target system that has wider significance for understanding and intervening in the world.

This diversity means that decisions taken at each stage are grounded on different ways of valuing the data, arguably resulting in the employment of criteria for selecting what counts as data that only partially overlap with each other. To biologists, data are objects that accurately capture plant properties at the time of recording. To technicians, data are objects that have an adequate degree of resolution and image quality. To data managers, data are objects that can be transferred between servers and fit standard formats for sharing through digital infrastructures such as databases. To computer scientists, data are objects that are tractable via automated analysis. These judgements are affected by a variety of factors, ranging from the ethos of relevant communities, standards for adequate specimens and images, conditions for data access and use, and researchers’ understandings of data ownership and value. What matters for my purposes is that the judgements involved in data processing go well beyond what Suppes and others call “data reduction” - a notion that assumes that what counts as data never changes, and all that data processing is doing is to narrow the focus of researchers from all the “raw data” garnered through experimentation to a smaller pool of “clean” data judged to be appropriate – and appropriately visualized - for analysis.

By contrast, I argue that a close look at data processing practices demonstrates that the diverse concerns to be found at each stage affect the very nature of what researchers view and treat as data. In line with these observations, in previous work I have proposed to adopt a relational approach to data epistemology within which data are defined in terms of their function within specific processes of inquiry. Thus, what defines data are ascriptions of evidential value, rather than assumptions about a fixed representational value which has to be somehow uncovered: “any object can be considered as a datum as long as (1) it is treated as (at least potential) evidence for one or more claims about the world, and (2) it is possible to circulate it among individuals/groups” (Leonelli 2016).

### Representation is not always the primary goal

The relational approach to data epistemology does not deny that data can be endowed with a representational function. Indeed, taking (some aspect of) data to represent a given phenomenon is necessary for data to function as evidence for a specific knowledge claim. The value of data as representations can, however, vary depending on which phenomenon they are associated to; and viewing a given set of objects as representations of a specific phenomenon is not a necessary nor a sufficient condition to their identification as data (potential evidence for claims) in the first place, nor to their use within (at least some stages of) the research process. In stage 1–5 above, we have seen how researchers are not centrally concerned with linking the ways in which data are produced and handled with more or less conceptualized features of the target system under investigation. Often such links lurk in the background, as in the case of stages 1 and 2 where setting up the material circumstances of imaging clearly involves a concern with producing imaging data that document the root system broadly conceived. However, the central aim of technicians, data managers, imaging specialists and computer scientists – particularly in stages 3 to 5 - is securing the *reliability, integrity, reproducibility and portability of data*. The focus is thus on the quality of images, design, computing requirements, care for plant specimens, the running of experiments and the accessibility of outcomes. Researchers are primarily concerned with making the objects generated via experiments *usable as evidence*, no matter which specific knowledge claims such evidence will be used to support*.* And indeed, while data processing affects the ways in which data are subsequently interpreted including their representational value, much of the work done on data during stages 1–5 is not meant as an act of interpretation and does not necessarily teach researchers anything about the target system under investigation.

In stages 6 and 7, by contrast, the reliability of data is largely assumed. The focus shifts towards identifying specific aspects of root-soil interactions that can be analysed using plot charts to regiment filtered imaging data, and evaluating whether and how the charts can work as an adequate representation of the aspects thus identified. This process helps to define the very phenomena of interest. Root width becomes the focus of investigation because it can be easily harnessed from imaging data and, thanks to the standards set up by technicians in stages 1 and 2, it can be reliably compared across plants and experimental runs. In stages 6 and 7, questions around how to interpret data as representing a specific feature of the target system take central stage. Researchers scrutinize the extent to which data support claims about specific phenomena, which is tied to the ways in which data are formatted and ordered at that specific moment of inquiry.^22^ Hence researchers typically need to develop: means of clustering and visualising data so that the data can be linked to a phenomenon of interest (for instance, by working with computer scientists to modify graphs such as in Fig. 6 to obtain a visualisation that documents a pattern of interest in ways that help to characterise root growth); and warrants through which that link can be justified and made plausible (for instance, by producing explanations of that pattern that build on existing knowledge of grass development and metabolism).^23^ What precisely data are taken to represent, and under which conditions, is thus the result of researchers’ decisions on what aspects of the target system are plausibly documented by the properties of the data clusters at hand. Whenever researchers take such decisions, I argue, they are using data as data models.^24^ I therefore define data modeling as the attempt to *learn about the world by analysing what a specific way of ordering and visualising data can teach about one or more aspects of a target system*.

### Data do not have fixed representational value

A crucial objection to this account consists of insisting that the way data are – their material features - determines what representational value is eventually bestowed upon them; and inferring from this observation that it is implausible and unwarranted to regard data as anything other than representations of the part of the world which they are intended to document. When considering the case at hand, this objection can be reframed through the following questions: could images such as that reproduced in Fig. 3 be taken to represent anything other than plant roots? Are these data not retaining a sort of “minimal aboutness”, an unbreakable connection to specific parts of the world which signals them as documents of plants and plants only?^25^ After all, as I already noted, stages 1 and 2 of data production are all about making sure that the images provide an adequate likeness of plant roots. This objection is particularly striking in the case of photographs, which is why I chose this type of object, rather than more abstract ones such as numbers, as an exemplar of biological data.

My response is to note that acknowledging the causal connection between the physical properties of the world and those of the objects used as data does not necessarily warrant an understanding of data as representations of the world. It is certainly true that data are objects produced through an interaction between humans and their environment, an interaction that in the case of research data is explicitly aimed at capturing and reproducing some of the world’s physical features. The material features of the data are strongly constrained by the physical features of the world – in other words, “the way the world is” is what enables data to have certain characteristics, whether these consist of the numerical values obtained via measurement or the shapes captured by a photograph. Philosophers such as Ian Hacking (1992), Hans-Jörg Rheinberger (2011) and Adrian Currie (2018) have referred to data as “marks” or “traces” precisely to signal the causal link between the conditions under which data are generated and the product of that interaction. What their work makes clear, however, is that “the way the world is” is not the only cause involved in the making of objects used as data. As in the case of plant imaging chambers, interactions between researchers and their environment are often heavily technologically mediated and carefully choreographed. Instruments and environmental conditions are thus also among the causal factors that determine the material features of the objects produced through those interactions. This makes data into objects that embody and document a particular kind of interaction with the world, rather than the world in and of itself. Furthermore, we have seen how the material features of data can be modified through data processing, thus further amplifying the set of causal interactions that data can be used to document. In this sense, data do have a “minimal aboutness” – but what they are about is a process of inquiry, rather than a specific aspect of the world taken in isolation.

This significantly expands the scope of the representational value that can be attributed to the data. It is definitely not the case that any object can be taken to function as data for any phenomenon. Depending on the nature of the inquiry at hand and the objects being considered as data, the material features of data may well pose strong constraints on the representational value eventually bestowed upon them. They do not, however, determine it fully: the representational value of the data needs to be evaluated in light of several factors beyond their material features, including those involved in both their production and their processing. In our example then, the shape, structure and topological properties of images such as Fig. 3 make them particularly well-suited to documenting plant morphology, which is not surprising since the technology used to generate the images is geared towards producing objects whose features make that aspect of the world amenable to investigation. In this sense, the data can be rightly viewed as being broadly “about plants”; and yet it remains possible to use them to represent phenomena that have nothing to do with plants and everything to do with the technology and agency involved in their production and processing. For example, the images could be used as data for a study on photographic techniques or on the applicability of Matlab software – in which case they would be interpreted as representing an imaging technique or a target for computational processing, and their capacity to represent an organism of features thereof would become irrelevant.

Another objection to this view consists of questioning whether it does in fact differ from Suppes’. My analysis of the differences between stages 1–5 and 6–7 of data processing could be interpreting as fitting precisely Suppes’ account, with data undergoing a series of manipulation and cleaning processes at the start of research and becoming a data model as soon as they are quantified and visualized through the employment of computationally enabled statistical tools. Aren’t the non-filtered images of plants in my case a clear example of “raw data”, and the plot charts an obvious instance of a data model in Suppes’ terms?

There are at least two reasons to answer this question in the negative. First, it is not the unfiltered images of stage 2 that end up counting as “raw data” from which charts are extracted. Rather, it is the filtered images obtained by stage 5 that are used as evidential ground for the construction and subsequent interpretation of graphs. Such data can hardly be called “raw” given the extensive processing I detailed in stages 3 and 4, which is not primarily conducted through statistical reduction and curve-fitting. Second, the preference for using filtered images as evidence does not extend to all research situations. For instance, unfiltered images may be preferable when investigating the mechanisms responsible for the appearance of an unusual trait in one of the plants (such as, for instance, a brown and jagged leaf). In such a case, the stipulations made by computer scientists in stages 3 and 4 are no longer applicable, and researchers need to evaluate anew which features of the photograph (and stage of cleaning/filtering) may constitute evidence – thus changing what counts as relevant data. The plot charts used as a data models in our case could well be used as “raw data” within a different research situation. This happens in part B of the SureRoot project itself, where plot charts are used as data from which visualization of roots counts across different environments and species can be extracted (Humphreys et al. 2018). Researchers involved in assessing whether an unusual trait is the result of an infection or a mutation may even treat (parts of) the plant specimens themselves as data; for example, the leaves of a given group of plants can be used as evidence for claims such as “while *Festulolium* leaves are typically green at an early stage of development, it is possible for specimens to produce brown leaves”. By the same token, what is taken to function as a data model can also vary depending on the situation of inquiry. Whole plant specimens can be used as data models in the sense of presenting an arrangement of data that functions as a representation of a particular phenomenon (e.g. a green *Festulolium* plant representing normal plant development).

In short, decisions about which of the activities examined in this case should be interpreted as instances of modelling can only be taken in relation to the research situation at hand. While Suppes’ view is tempting in its simplicity and elegance, I argue that it underestimates – and ends up hiding away - *the potential of the objects produced by researchers in a given situation to function as data or data models in other situations of inquiry*. I contend that this potential is crucial to the epistemic power of scientific practices of data selection, processing and ordering, and has important ramifications for scientific epistemology – not least, for understanding how researchers identify phenomena in the first place, as I discuss in the next section.

### Data processing defines the evidential space within which phenomena are identified and stabilized

I already mentioned how procedures of data processing and modelling can be used to identify the phenomena of interest in any specific investigation – a situation typical of exploratory experimental research. I shall now consider this claim in light of the proposed functional distinction between data and data models.

In our empirical case, it is clear that decisions made early on in the production of *Festulolium* images have an impact on which phenomena are ultimately identified as the focus of the investigation. While the initial target of inquiry was broadly conceived as the root system as a whole, decisions made in stages 2 and 3 restricted the scope of inquiry. For instance, taking photographs only twice per day makes it impossible to use the images as evidence base for claims about the fine-grained development of leaves (which would require more fine-grained documentation of how leaves develop over 24 h). The choice of root width and position as key parameters for stages 4 and 5 further narrowed what I shall henceforth refer to as the *evidential space* within which images can be used as data: that is, the range of phenomena whose analysis data could plausibly serve as evidence.

Nevertheless, the researchers involved in data processing are not always concerned with the implications of these decisions on the target of the study. This is only one of several factors – including material and technical constraints - that researchers need to take into account. Unsurprisingly, it is at the point of ordering the data that discussions about what is actually being represented acquire central stage. In stages 6 and 7, researchers actively question the extent to which a given data arrangement can provide information about a particular aspect of the root system, and it is then that they may choose to revisit previous stages of data processing so as to modify the evidential space thus defined. Those discussions are at once constrained and validated both by the physical features displayed by data at that stage of processing, and by the extent to which researchers can match the characteristics of the data cluster/model that they produced with specific claims about phenomena.

**Fig. 7:**
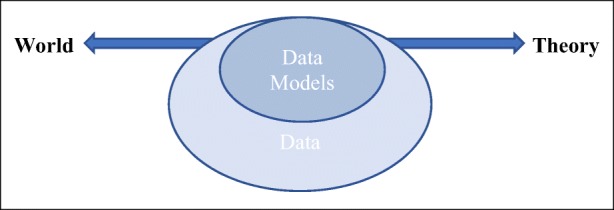
Illustration of a key difference between the relational view of data models defended here and the representational view depicted in Fig. 2. Decisions about what counts as data define the evidential space within which representational claims grounded on such data can be formulated and corroborated. Data models are developed within this evidential space in order to represent specific phenomena

Hence, both data processing and data ordering contribute to carving out what phenomena researchers are actually able to produce knowledge about. Going one step further than data processing, modeling marks the situations where researchers explicitly discuss the value of data as evidence for specific representational claims – which requires the identification and stabilization of the phenomena featured in those claims. This tells us something significant about why data models are such an important component of processes of inquiry. Data models are where evidential and representational considerations meet: where researchers consider the evidential value of a particular way of ordering data vis-à-vis the assumptions and commitments made in the course of producing and processing such data. Accordingly, the representational value of data models can be conceptualised as a subset of the evidential space delimited by the ways in which data are processed. As illustrated by Fig. 7, data models are clusters of data that are accepted as (1) useable evidence and (2) representing a particular phenomenon in ways that help generate new knowledge about the world.^26^

My analysis mirrors insights already garnered by philosophers on the processes through which phenomena are singled out within experimental research, a discussion that found new vigor through Bogen and Woodward’s (1988) and Hacking’s (1992) seminal analyses of the skills and procedures involved in inferential reasoning from data. While Bogen and Woodward emphasized the empirically grounded nature of such inference, James McAllister (2010) depicted it as largely arbitrary and theory-laden, due to the large number of equally plausible patterns which any dataset can be taken to indicate. Adding to like-minded arguments offered by Michela Massimi (2011) and Paul Teller (2010) among others,^27^ my view offers a middle-ground between these two alternatives. While data models necessarily reflect the theoretical commitments and goals of researchers using them (as emphasized by McAllister), they are also strongly constrained by the empirical conditions under which data have been processed (as argued by Bogen and Woodward), which delimit the evidential space within which representational claims can be stipulated and phenomena can be identified and stabilized.

Yet another way to emphasize this point is provided by Uljana Feest’s (2011) distinction between the skills required to identify phenomena emerging from data patterns (which she calls “surface regularities”) and those underpinning the identification of unobservable and highly conceptualized phenomena (which she calls “hidden regularities”), a move that runs parallel my own distinction between the concerns and skills involved in processing vis-a-vis modelling data. Feest concludes that “neither of these two kinds of phenomenon can be stabilized in isolation. [..] what is stabilized when phenomena are stabilized is the fit between surface regularities and hidden regularities” (Feest 2011, 57). Data models in my account have precisely the function of facilitating such a fit. They mark the stage of research where scientists interrogate the relation between data processing procedures, which delimit the evidential space and the type of surface regularities that can be considered in their investigation, and data modelling choices, which specify which aspect (“hidden regularity”) of the target system the data are taken to represent – and thus, which phenomena researchers will produce knowledge about. Data models are thus crucial facilitators of the iterative interplay between empirical constraint and theoretical interpretation, a situation variously described by Paul Edwards (2010) as a “data-model symbiosis” and Rheinberger (2015, 324) as a “cyclical feedback” between data and models. As Harris noted in relation to data processing in physics, “a description in terms of data models facilitates improved understanding of the interplay between theoretical principles, theoretical interests of scientists, and aspects of the data model that are due to experimental constraints imposed by nature” (Harris 2003).

**Fig. 8:**
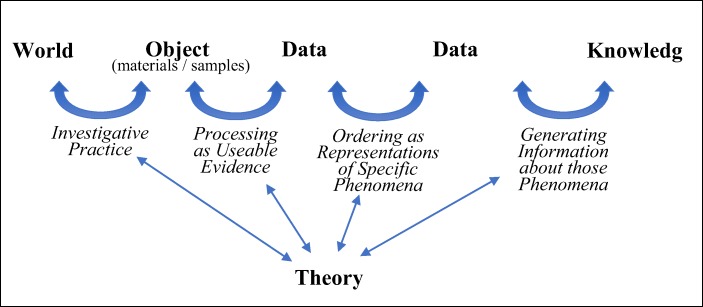
A graphical representation of the relational view of data and models within empirical research. In contrast to Fig. 2, this linear representation illustrates how “world” and “theory” are no longer on a continuum, but rather theory affects the decisions taken by researchers around what objects are generated through research, which of those objects count as data, which data clusters can be used to represent specific phenomena (and thus amount to data models), and what knowledge is obtained about those phenomena. Arrows go both ways to illustrate the iterativity between stages

Ultimately, in distinguishing between data processing and data modelling, I am highlighting the different extents to which theory - understood broadly as a set of theoretical commitments and goals - impinges on inferential processes from data. This brings me even further away from the representational view discussed in section 1, within which research components such as data and models are what justifies and validate specific theoretical interpretations of the world. If we acknowledge that data processing and modelling play important and distinct roles in the identification and stabilisation of phenomena, the very conceptualization of inquiry as a set of tools linking “world” and “theory” no longer holds. Rather, empirical inquiry is best depicted as an iterative process consisting of four key steps, represented in Fig. 8: (1) the production of objects of investigation through interaction with the world; (2) the processing of such objects so that they can function as data, which unavoidably involves a restriction in the evidential space within which data can be credibly used; (3) the ordering of data through data models, so that they can represent specific phenomena; (4) and the use of data models to develop knowledge claims about those phenomena.

As Fig. 8 emphasises, theory is involved in each of these steps. What the figure does not express, however, is that theory can take a variety of different forms at different stages of inquiry. In steps (1) and (2), which affect what ends up counting as data, theoretical commitments are not necessarily articulated in the form of high-generalisations or coherent conceptual frameworks. They can be incorporated in the choice of materials and samples, experimental instruments and data sharing procedures – the ways, in other words, in which researchers carve nature’s joints and thus limit the conceptual space within which data can be used as evidence.^28^ Steps (3) and (4) are where researchers actively question, identify and stabilise conceptual assumptions about the nature of phenomena to be investigated, which shape the content and formulation of the knowledge claims being produced. Generalisations and law-like statements are more likely to be explicitly acknowledged and to play an active role here.

## Conclusion

How do we avoid a view of science as models all the way down? I argued that this involves paying attention to data processing practices and the ways in which they underpin the attribution of representational value to specific arrangements of data. Curve fitting is not what defines a data model and the degree of human manipulation does not define what counts as data either. Rather than portraying the difference between models and data as a difference in intrinsic properties, format or levels of abstraction, I proposed to view it as a difference in the interests and evaluative criteria of the researchers using them, which in turn determine their epistemic role.^29^

Data are results of interactions between researchers and the world, which are construed and processed to function as usable evidence for claims about phenomena. As in the case of phenotypic images, they are objects that researchers are interested in treating and evaluating as evidence for claims about a target system (a whole plant). The very process of defining what constitutes acceptable evidence helps researchers to narrow their investigative focus to specific phenomena of interest within that target system (e.g. root width, growth, molecular composition). Procedures of data production and processing thus define the evidential space within which representational claims can be made in the first place, and in so doing, they affect which features of a given target system are investigated within a given line of inquiry. The broader the evidential space traced by data, the more re-usable data are for different investigative purposes.

By contrast, data models are ways of ordering data that are evaluated, manipulated and modified with the explicit goal of representing a phenomenon, which is often (though not always) meant to capture specific aspects of the world. Data models help to identify and clearly the phenomenon of interest in any one investigation, while at the same time highlighting how a given dataset can be convincingly used as evidence for claims about that phenomenon.^30^ Unlocking the informational content of data is only possible when aggregating them and interpreting them as representations through data models. Notably, this representational function is compatible with data models helping to do many other things too, such as predicting, describing or explaining. What matters for my purposes is that these additional functions are predicated on data models having representational value, whether or not the representation is taken to instantiate the properties of entities in the world.^31^

My analysis shows that it is possible to distinguish between research activities primarily concerned with making data useable as evidence (no matter for which claim) and activities primarily concerned with using data as evidence for a specific claim. Whether a set of objects functions as data or data models is defined by their role in specific epistemic activities, and particularly the ways in which they support the identification and characterization of the targets of investigation in any one situation of inquiry. Data processing is about establishing the reliability and usability of given objects as documents for an empirical target system, which also defines and delimits the evidential space within which research can be reliably conducted. Data modelling is about identifying the evidential value of given data clusters with respect to specific claims about a target system. This captures the concerns with the procedures and motivations for data processing that Suppes was well-aware of and preoccupied with, without however needing to posit a fixed hierarchy of data and models or a highly exclusionary definition of what counts as data models. The latter becomes a functional issue relating to the use of data as models to represent a specific phenomenon, rather than a structural issue relating to the physical properties of data themselves. As van Fraassen similarly pointed out in relation to representational practices, “the very same object or shape can be used to represent different things in different contexts, and in other contexts not represent at all” (2008, 27), and whether or not data are taken to represent depends on the specific situation and stage of research: “measurement outcomes are at a certain stage to be conceived of as trading on selective resemblances” (ibid., 91).

**Fig. 9:**
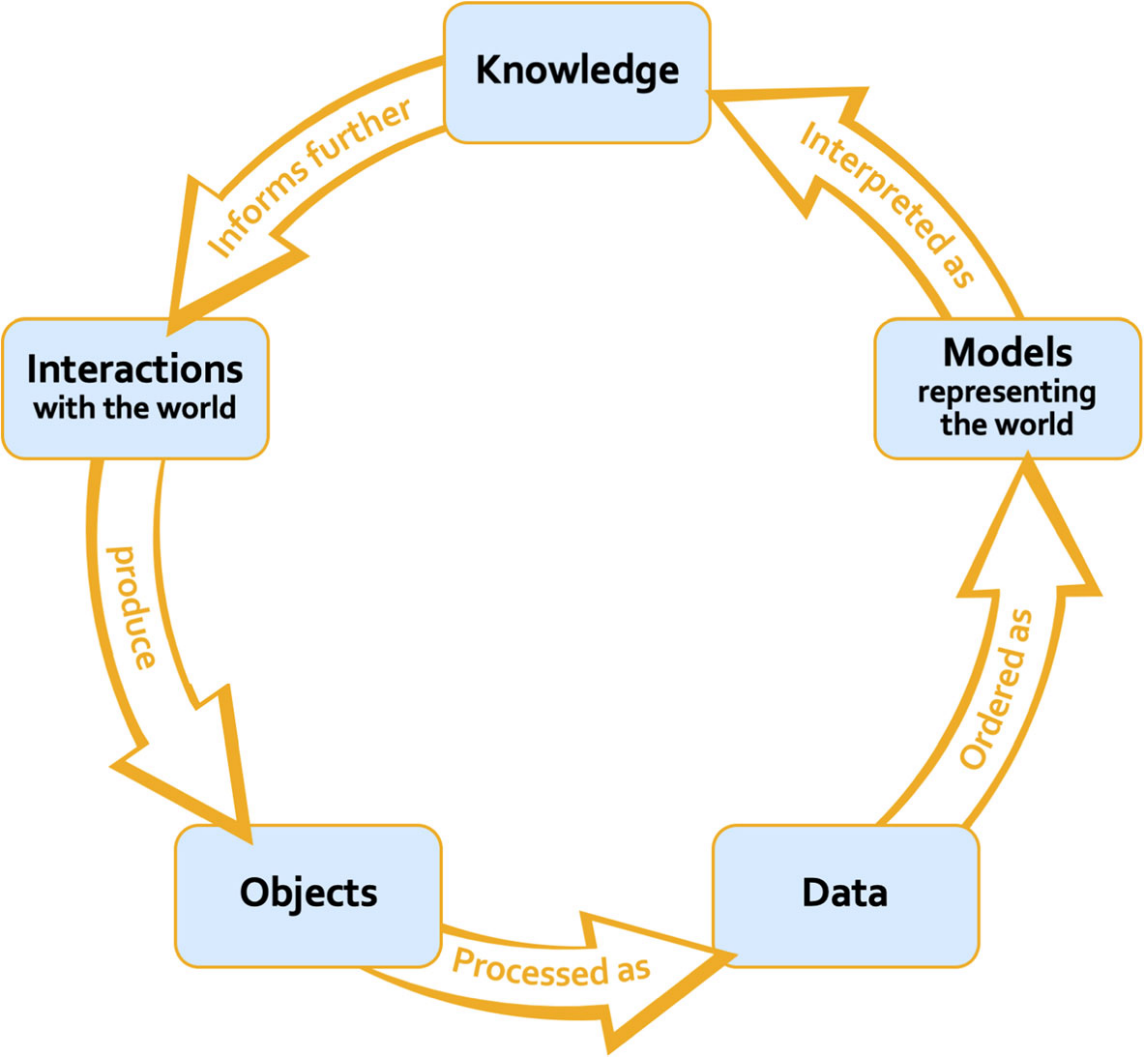
A cyclical representation of the process of empirical inquiry grounded on the relational approach to data and models

This framework constitutes a viable alternative to Suppes’ hierarchy of models in at least four ways.^32^ First, it accounts for situations of empirical research in which statistical methods are not the sole/primary means of validating data, phenomena of interest are not stabilised at the start of the inquiry, and data do not consist exclusively of numerical quantities. Second, it does not rely on uncritical appeals to the hazy notion of “raw data”. Third, it recognises that data and data models have no ontological fixity – and that by assuming that they remain the same throughout inquiry, philosophers lose sight of the importance of data processing procedures in defining the range of phenomena for which data can be used as evidence (i.e. the evidential space). Fourth, it provides a definition of data model that emphasises the extent to which theoretical expectations and interest in particular aspects of the target system intersect with the empirical conditions under which research is carried out.^33^ More broadly, this account of data models recognizes the intertwining of data acquisition and interpretation in research practice noted by Harris (2003) and does justice to the significant epistemic role of data processing activities. Rather than as a hierarchy, the process of empirical inquiry can be conceptualized as a cycle in which the objects obtained through investigative practices are processed as data, the data are ordered into models, and the models are used to produce knowledge which in turn inform further investigative practices (Fig.9).

## Footnotes

1 On the characteristics of exploratory research and its contrast to hypothesis-driven modes of inquiry, see Steinle (1997), Burian (1997), Waters (2007) and O’Malley (2008).

2 The extent to which computational tools intersect with qualitative judgements on what counts as data and how they should be used is particularly significant at a time where the development of automated methods for large-scale data production and analysis are hailed by some analysts and many funding bodies as the next frontier of artificial intelligence (e.g. Mayer-Schönberger and Cukier 2013).

3 My analysis is compatible with a wide spectrum of views around what representation involves in relation to data and modelling, including accounts within which models represent non-existing (e.g. abstract or fictional) entities and the target of representation is not mind-independent. I am particularly sympathetic to the liberal but precise account of how material models represent through denotation, exemplification, key and identification by Frigg and Nguyen (2018).

4 The project was funded by the UK Biotechnology and Biological Sciences Research Council LINK grant BB/L009889/1 (which is match-funded by the UK grassland industry sector).

5 This empirical analysis results from an extensive investigation of data processing activities across biological research sites in the UK which I conducted between 2015 and 2018. This involved visits to the sites in which data were produced, stored, disseminated, visualized and analysed, where these research activities were documented via ethnographic observation, photographs and videos; a review of the resulting scientific publications and the ways in which authors describe their methods and techniques therein; the study of websites and digital databases used as reference points or tools throughout the inquiry; and in-depth, semi-structured interviews with the researchers involved, including technicians as well as software and computer experts in charge of processing and storing data. The point of gathering such diverse and rich information on data processing was to document the nuances and sophistication of related activities, resulting in thick descriptions of the stages through which data of various types are processed for the purposes of scientific discovery, such as in the case analysed within in this paper. More information on this method and overarching project can be found on the Exeter Data Studies website (URL: www.datastudies.eu, accessed October 2018). Selected interview transcripts and photographs pertaining to the NCPP case are available as open data on Zenodo (URL: https://zenodo.org/communities/datastudies, accessed October 2018).

6 As pointed out by Woodward in 2000, the evidential relationship between data and claims is conceptualized as “a purely formal, logical, or a priori matter” – a point highlighted and critiqued also by John Norton (2010), whose theory of material induction emphasizes the role of local facts in warranting inductive inferences.

7 I provided an extended critique of the representational approach to data in Leonelli (2016).

8 Reiss (2015) provides a useful analysis of this situation.

9 I have previously noted how, despite providing a revolutionary analysis of data processing that countered the view of statistics as sole arbiter of inference from data and inspired my own views, Bogen and Woodward (1988) do not explicitly take issue with the representational account of data (Leonelli 2016).

10 I will not linger on the problematic nature of Suppes’ hierarchy in this paper, which has already been critiqued by Koray Karaca (2018) with reference to data processing procedures in particle physics.

11 Mayo clearly acknowledges that “modeled data, not raw data, are linked to the experimental models. Accordingly, two questions arise [..] how to generate and model raw data so as to put them in the canonical form needed to address the questions in the experimental model [..] how to check whether actual data satisfy various assumptions of experimental models” (Mayo 1996, 129). Her work masterfully shows how statistics can be employed to help with these questions, but does not explore whether and how researchers may address these questions beyond the application of statistical techniques – which is my main concern here.

12 See for instance Suppes (1997, 2003).

13 For a rich discussion of the history of phenotyping experiments, see Taylor and Lewontin (2017). For a review of current developments in phenotyping and the significance of data and data analytic tools within them, see e.g. Bolger et al. (2017) and Coppens et al. (2017).

14 In the words of Principal Investigator Mike Humphreys, “the study represented a real breakthrough in high throughput root analysis necessary for future grass breeding.. helpful to new holistic approaches to crop improvement that take into account not only measures of crop production but the impact these have on their surrounding habitat” (pers. comm., October 2018; see Humphreys et al. 2018).

15 The following video gives a good overview of what the NCPP smart glasshouse looks like and how it works in practice: https://www.youtube.com/watch?v=8qBsVP0j70k. Smart glasshouses are increasingly popular experimental spaces for phenotyping around the world, though their characteristics and the extent to which procedures are automated and insulated from the external environment varies considerably (George et al. 2014). It should be noted that the majority of plant specimens used for SureRoot was housed outside the glasshouse prior to screening, so as to maximize the plants’ exposure to the natural environment while still carefully monitoring and measuring their exposure to light, water and humidity. As the majority of experiments processed in facilities such as NCPP rely on specimens grown exclusively in the glasshouse, it remains important to stress the significance that environmental controls inside the glasshouse have for subsequent imaging and the interpretation of the data.

16 Many studies of root growth avoid this source of variability by resorting to soil-free growth media (which have the further advantage of being transparent, thus considerably facilitating the imaging).

17 NCPP has an alarm system to signal overnight blockages, but somebody comes in to check only if the whole system is compromised. Experiments typically run from midnight to 11 am and from 3 pm to 10 pm, so there is only a small window for maintenance, which is sometimes taken up with other experiments. Technicians are concerned about the “lack of redundancy in the system”, which is likely to worsen as the apparatus ages.

18 The SureRoot experiment involved only 28 plants, with each round of imaging taking 1 h and 40 min. For experiments involving a larger group of plants, imaging can take as long as 12 h.

19 For a conceptualisation of the epistemic function of “dirt” in the production, cleaning and visualization of data, see Boumans and Leonelli (under review).

20 When I visited, the name of each file would indicate the plant type, mutant number, experiment number and repeat number, thus including a lot of information about the circumstances of data production. The labelling system was, however, far from standardized and the keys to the system were not written down anywhere which made the files difficult to handle computationally and created problems of scale for very large experiments. Such circumstances vividly illustrate the significance of a data manager’s know-how towards the re-use of data, though such expertise remains equally important in situations where commonly used standards need to be applied to locally generated data (e.g. Boumans and Leonelli under review).

21 During my visit at NCPP, such decisions were constrained by the lack of a systematic approach to data storage (which was all done via local servers) and of a data sharing policy for the whole institute, which were in turn due to a shift in personnel: the previous data manager had just left, bringing with him a wealth of local knowledge that the new data manager had yet to reconstruct.

22 Notably, this rarely involves focusing on a single datum: researchers typically need to consider groups of data, whose size and degree of homogeneity can of course vary. For a detailed discussion of this issue, see Leonelli and Tempini (under review) and particularly the chapters by Mary Morgan and James Griesemer.

23 On the use of warrants to enhance the plausibility of evidential reasoning, see Chapman and Wylie (2016, chapter 1), who in turn build on Toulmin’s argumentative schemas (1958).

24 Note that the data model in this case does not need to be interpreted as representing by directly instantiating properties of the root system. Representational value can rather be interpreted as the ability of the model to signal factors that may help explain the structure and behavior of the root system relative to the environmental conditions at hand. As noted by Frigg and Nguyen (2016), “representation is a much broader notion than mirroring”.

25 I am grateful to an anonymous referee who spurred me to clearly frame and address this objection.

26 Note that my discussion focuses on data models as the “meeting point” between data and all other types of models used within empirical inquiry. I regard this view of data models as compatible with the recognition that other types of models have different characteristics and epistemic functions, and may be much closer to the “theory” end of the spectrum depicted in Fig.7 (as in the case of some theoretical and mathematical models). A rigorous evaluation of this claim is required, yet this lies beyond the scope of the present paper.

27 Massimi (2011) defines phenomena as ‘conceptually determined appearances’, while Teller insists that “what will count as a phenomenon appears to be tangled up with the ubiquitous use of idealizations in science” (2010, 820). Indeed, Teller’s distinction between “phenomenon type” and “phenomenon token” complements my relational account: the identification of phenomena types is significantly affected by the choices made by researchers about what objects count as evidence, while the identification of phenomena tokens depends largely on the use of data within data models as representations of a specific aspect of a target system. Other sympathetic accounts include Bechtel’s study of the role of mechanistic reasoning in shifting conceptualizations of phenomena (2017) and Miles MacLeod’s and Nancy Nersessian’s work on “adaptive problem spaces” within which phenomena are identified (2013).

28 In Leonelli (2016), I describe this type of theorizing as “classificatory theory”.

29 I thus move away from structural accounts of empirical modelling, taking heed from Frigg’s argument that: “one has to recognize that structures cannot be connected to anything in the world without the mediation of non-structural concepts, and attempts to bypass this conclusion by appeal to data models fail” (2010, 111).

30 This characterization of data models is compatible with recognizing that such models can themselves fulfil a variety of epistemic functions, ranging from description to prediction, and can be grounded on a wide spectrum of ontological commitments (and intensity of ontological commitments).

31 My account is thus also compatible with the recognition that data models can be grounded on a variety of different ontologies and on different intensity of ontological commitments.

32 This position expands and refines my arguments in (Leonelli 2016), resulting in a more critical take on Suppes’ hierarchy of models (which I had previously argued to be broadly compatible with my relational account).

33 This account of data models thus aligns with the blossoming philosophical scholarship defending a functional understanding of modelling as a situated activity (e.g. Morrison 2009); as well as the approach to measurement outlined by Eran Tal and Wendy Parker. Indeed Parker’s (2015) view that “measurement is relative to a theory or model, and the outcome of a measurement is *a selective representation of the entity measured*” works in the framework I proposed, as long as the outcome of the measurement is regarded not as data themselves, but as a data model. As noted by Tal (2013) and Floridi (2011), checking the consistency and accuracy of measurement outcomes in relation to a given target system is a fundamental part of data quality assessment.
